# Explainable artificial intelligence-based classification of asthma and COPD from xenon-129 ventilation magnetic resonance imaging

**DOI:** 10.1183/23120541.00234-2026

**Published:** 2026-10-19

**Authors:** Joshua R. Astley, Laurie J. Smith, Alberto M. Biancardi, Rod Hughes, Rod Lawson, Bilal A. Tahir, Helen Marshall, Jim M. Wild

**Affiliations:** 1 POLARIS, Section of Medical Imaging and Technologies, Division of Clinical Medicine, School of Medicine and Population Health, The University of Sheffield, Sheffield, UK; 2 Insigneo Institute, The University of Sheffield, Sheffield, UK; 3 Early Development Respiratory Medicine, AstraZeneca, Cambridge, UK; 4 Sheffield Teaching Hospitals NHS Foundation Trust, Sheffield, UK

## Abstract

**Background:**

Diagnosis of asthma and/or COPD is challenging due to overlapping clinical and physiological characteristics; however, due to varying treatment recommendations, determining the correct diagnosis is vital to clinical care. Hyperpolarised xenon-129 ventilation (^129^Xe)–magnetic resonance imaging (MRI) provides high spatial resolution three-dimensional images of gas distribution within the lungs. Recently, artificial intelligence (AI)-based models have successfully classified pulmonary pathologies *via* medical images, but due to their black-box nature, clinical application is challenging. Here, we aim to develop an explainable AI framework to classify patients with asthma and/or COPD using ^129^Xe-MRI alone.

**Methods:**

^129^Xe-MRI from 207 patients with asthma and/or COPD were classified using a vision transformer (ViT) and a convolutional neural network (CNN). Regional visualisations of areas of importance (AOIs) associated with each diagnosis were generated. Regional patterns were investigated to develop a shape-based metric which quantified asthma-like and COPD-like features.

**Results:**

The ViT accurately classified asthma and/or COPD patients in 83% (n=25/30) of cases (area under the curve (AUC) 0.86, F_1_ score 0.82), outperforming the CNN (AUC 0.71, F_1_ score 0.62). Patients with asthma had more diffuse, elongated AOIs associated with disease grouping while patients with COPD had more focal, spherical AOIs. The proposed sphericity score weakly correlated with conventional lung function metrics in clinically understandable ways. When applied to patients with overlapping features of asthma and COPD (asthma+COPD), the classification framework informed the prevalence and regional distribution of asthma-like and COPD-like features.

**Conclusion:**

Explainable AI classification of ^129^Xe-MRI could facilitate diagnosis of patients with asthma and/or COPD, including determining the prevalence of asthma-like or COPD-like features in patients with asthma+COPD, potentially informing management and treatment.

## Shareable abstract

**Asthma and COPD were classified from ^129^Xe MRI using a 3D vision transformer. Explainable AI derived a novel sphericity-based metric that is sensitive to detecting regional asthma *versus* COPD-like patterns beyond conventional lung function metrics.** https://bit.ly/4ag0Gez

## Introduction

Patients with asthma and/or chronic obstructive pulmonary disease (COPD) experience lung disease with overlapping clinical and physiological characteristics [1]. Conventional criteria poorly differentiate between the diagnostic groups of asthma, COPD and asthma+COPD [2]. Ensuring the accurate and timely diagnosis of these heterogenous conditions is essential due to varying treatment recommendations between conditions [1, 3, 4]. For example, long-acting bronchodilators alone are recommended for patients with COPD but are contraindicated for patients with asthma [5]. Between 15% and 32% of patients with asthma or COPD exhibit features of both conditions [3] and determining if these patients with asthma+COPD have more asthma-like or more COPD-like disease could help guide management and treatment.

Hyperpolarised xenon-129 ventilation (^129^Xe)–magnetic resonance imaging (MRI) enables the visualisation and quantification of regional lung ventilation with high spatial resolution [6]. Whole-lung metrics derived from ^129^Xe-MRI have shown significant differences between patients with asthma and COPD [7, 8]; these include the ventilation defect percent (VDP), a measure of how much of the lung is ventilated, and the ventilation heterogeneity index (VHI), a measure of the heterogeneity of the ventilated airspaces [9]. Patients with COPD had less and more heterogeneous ventilation than patients with asthma and, to a lesser extent, patients with asthma+COPD [8]. However, disease classification of individual patients from ^129^Xe-MRI metrics alone is unfeasible without data from other imaging, pulmonary function and clinical metrics. Notwithstanding, ^129^Xe-magnetic resonance (MR) ventilation images are information-rich, greyscale spectrums of signal that provide detailed information on regional structures, information which is not fully captured by existing ^129^Xe-MRI metrics.

Convolutional neural networks (CNNs) have been used for the classification of pulmonary pathologies, including lung cancer [10] and COVID-19 [11], becoming ubiquitous in medical image classification tasks. Recently, CNNs have been utilised for the classification of ^129^Xe-MR images of several pulmonary pathologies [12]. Advancements in artificial intelligence (AI) architectures, such as vision transformers (ViTs) [13], have demonstrated improved performance compared to CNNs [14], in a number of medical imaging tasks [15]. However, deep learning classification approaches lack interpretability, reducing trust in the predicted diagnoses. Techniques have been developed that provide insights into the network's predicted classification [16]. Occlusion sensitivity mapping (OccSens) provides a map of network attention by sequentially removing small regions of the scan and quantifying their impact on classification accuracy [17, 18].

Here, we propose a ViT-based approach that utilises ventilatory information in ^129^Xe-MR images to classify patients with asthma and/or COPD. We employ occlusion sensitivity mapping to provide regional areas of importance (AOIs) associated with each diagnosis group and propose a novel OccSens-derived shape-based metric to quantify the prevalence of asthma and COPD features in ^129^Xe-MR images.

## Materials and methods

The dataset contained a total of 207 ^129^Xe-MRI scans from patients with either asthma, COPD or asthma+COPD. 139 patients, including all asthma+COPD cases, had physician-assigned diagnoses in line with the NOVELTY observational longitudinal study (NOVELTY, NCT02760329) philosophy [19], whilst the remaining 68 patients were diagnosed according to guidelines [5, 20]. Patient data used in this work was pooled retrospectively from prospective clinical imaging studies and clinical referral cases. All prospective studies received ethical approval by the national research ethics committee with participants (or their guardians) providing informed written consent. Appropriate consent and permissions have been granted by the sponsors to utilise this data for retrospective analysis purposes. Some of these data have been previously published [8, 21, 22].

Patients were recruited from two primary care centres in York, UK and secondary care centres in Sheffield, UK and surrounding areas. All patients were assessed with ^129^Xe ventilation MRI and pulmonary function tests at the University of Sheffield. All participants underwent three-dimensional (3D) volumetric ^129^Xe-MRI acquired in the coronal plane at approximately functional residual capacity+inhaled bag volume (from 400 mL to 1.2 L) with full lung coverage at 1.5 T. Patients were scanned across two hospitals and on one of three scanners: a HDx, Artist or 450w GE scanner (GE Healthcare, Milwaukee, WI, USA) [23]. ^129^Xe was polarised on site using an in-house developed rubidium spin-exchange polariser [24]. Flexible quadrature radiofrequency ^129^Xe transmit–receive coils were employed with a 3D balanced steady-state free precession sequence [25] with the following settings: repetition time/echo time in the range of 4–6.7/1.38–2.2 ms, in-plane resolution of ∼4×4 mm^2^, slice thickness of 10 mm. A ∼40–48 cm field of view with a flip angle of 9° or 10° at a bandwidth of ±8 kHz or ±16 kHz was used.

### Deep learning experiments

In this work, we develop, evaluate and compare two specific deep learning models, namely, a CNN-based DenseNet model and a ViT attention-based model. Network development, training, inference and evaluation were completed using the open-source medical imaging and deep learning framework monai 1.3.0 [26] built on top of PyTorch 2.2.2. The following sections describe the preprocessing and data augmentation strategy as well as the network hyperparameters of each model.

#### Preprocessing and data augmentation

All scans were subject to preprocessing prior to network input; for the CNN model, scans were resized to 256×256×32 voxels and for the ViT model, scans were resized to 128×128×32 voxels. Input volumes were reduced for the ViT model due to the increased memory requirements during ViT training. For both models, image intensities were normalised to be between 0 and 1. On-the-fly data augmentation was employed to minimise overfitting and improve robustness; this included affine rotation and scaling augmentations with limits −12–12° and −10–10%, respectively. Horizontal flipping augmentations were also employed. All augmentations were randomly applied at a probability of 50% at test time.

#### DenseNet-121

A modified DenseNet-121 CNN [27] architecture, which utilises Dense blocks of increasing size (16, 32, 64 and 128) and three residual units, was trained with parameters based on a parameter search conducted on the validation set, resulting in a ReLU (rectified linear unit) activation [28], Adam [29] optimisation (α=1×10^−5^) and a cross-entropy loss function. A batch size of four was used for network training and batch normalisation was applied in each block. A final linear layer with Softmax activation at a fixed threshold of 0.5 was applied; raw logits were also recorded for the final output layer.

#### Vision transformer

A modified ViT architecture [13], which utilises a combination of CNN layers and self-attention blocks, was developed. The network contained 12 layers with 12 attention heads; each hidden layer has a dimension of 768 and each feedforward layer has 3072 dimensions. A patch size of 32×32×32 voxels was utilised to reduce computational requirements. A post-activation SoftMax activation layer was employed to generate predictions across the two classes. A parameter search was conducted on the validation dataset resulting in network hyperparameters of AdamW [29] optimisation and a cross-entropy loss function; a dropout of 0.1 was employed. A final linear layer with Softmax activation at a fixed threshold of 0.5 was applied; raw logits were also recorded for the final output layer

### Explainable AI framework

Due to the often-opaque nature of many deep learning models, explainable AI techniques have been developed to provide an understanding of how predictions are generated by a network; this is particularly necessary in medical contexts where decisions made without explanations are likely to be problematic.

In this work, we utilise a technique known as OccSens to understand which regions of the input scan are used in the resulting disease prediction [17, 18]. OccSens is a *post hoc* explainable AI technique where a range of predictions are created *via* multiple passes of the network. In practice, a section of the scan was first occluded, in this case a 32×32×32 three-dimensional cube, and then a classification prediction made. Subsequently, a sliding filter was applied with a 75% overlap to the previous occluded region and a new classification prediction made; this was then repeated across the whole scan, creating a heatmap which corresponds to regions which lead to lower and higher prediction accuracy. If an area of the scan is important to the network, then when this area is occluded, the resulting classification accuracy should reduce. Hence, this provides an understanding of which image regions are associated with each classification and, ultimately, provides a level of explanation of previously uninterpretable deep learning models.

### Data split and validation strategy

The dataset was divided into training, internal validation, testing and investigation datasets, representing four distinct datasets with no overlapping patients. The training dataset comprised 113 patients (asthma n=61, COPD n=52) and the internal validation dataset comprised 20 patients (asthma n=10, COPD n=10) which were used to determine the appropriate number of iterations; the CNN model was trained for a maximum of 40 epochs and validated every two epochs, whereas the ViT model was trained for a maximum of 200 epochs and validated every 10 epochs. Early stopping was employed to reduce potential overfitting by stopping training if the validation loss did not decrease for five validation cycles. The models with the highest accuracy on the internal validation set were selected for comparison on the testing dataset.

The testing dataset comprised 30 patients (asthma n=15, COPD n=15) and was stratified to ensure that the average VDP was equal between asthma (mean±sd VDP 9.45±6.93%) and COPD (mean±sd VDP 9.44±6.09%) patients, emphasising that any classification could not be based on disease severity. VDP values were computed from semi-automated segmentations, with any necessary manual editing of both functional and structural regions by several expert observers. However, these segmentations were only used for the VDP calculation, facilitating matching across disease cohorts, and were not used as a network input.

A final investigation dataset was also included, containing 44 patients with features of both asthma and COPD (labelled “asthma+COPD”), which was utilised as a clinical “testing ground” for unconfirmed cases.

### Development of an OccSens-based metric of asthma- and COPD-like features

An additional aim was constructed to quantify shape-based differences in OccSens maps, which could serve as a biomarker of asthma-like and COPD-like features, derived from the sphericity of 3D OccSens intensity maps. The proposed sphericity score aims to provide a singular metric that is indicative of a continuum scale spreading features of asthma and COPD. It measures how closely the most important feature in the 3D OccSens maps resembles a perfect sphere; practically, OccSens maps were generated for a given case and then the top 10% of intensity values were segmented using thresholding. Subsequently, the largest connected component was selected and maintained for analysis. The sphericity score (ψ) is thus defined in equation 1 as,

$$\psi =\frac{\pi^{\frac{1}{3}}{\left(6V\right)}^{\frac{2}{3}}}{A},$$

where the volume (V) is calculated based on the number of voxels in the largest component and the surface area (A) is approximated using a 3D surface mesh of this component [30]. A value of ψ between 0 and 1 was then generated for each asthma or COPD case in the whole dataset, exclusive of asthma+COPD cases. The sphericity was also computed for the top-three connected components for comparative sensitivity analysis.

### Analysis and statistical comparisons

Asthma and COPD predictions were evaluated using a confusion matrix with associated metrics, precision, accuracy, specificity, sensitivity, F_1_ score and area under the curve (AUC). Normality was tested using Shapiro–Wilks tests and the appropriate parametric or nonparametric test was conducted. Unpaired t-test of sphericity scores for asthma and COPD patients were conducted. p<0.05 was defined statistically significant. Pearson correlations between sphericity scores and functional lung metrics, transfer factor of the lung for carbon monoxide (*T*_LCO_), forced expiratory volume in 1 s (FEV_1_), VDP, VHI, the ratio of residual volume to total lung capacity (RV/TLC), and the COPD assessment test (CAT) score were calculated.

## Results

### CNN *versus* ViT comparisons

Patient demographics, including key functional lung metrics, are provided in table 1 for each disease group separately and the dataset as a whole. For the CNN model, the best performance on the internal validation dataset was achieved at 32 epochs with an AUC of 0.90. For the ViT model, the best performance on the internal validation dataset was achieved at 180 epochs with an AUC of 0.85.

**TABLE 1:** Patient demographics, pulmonary function tests and xenon-129 ventilation (^129^Xe)–magnetic resonance imaging (MRI) ventilation metrics

	Asthma	Asthma+COPD	COPD	Total
**Number of participants**	86	44	77	207
**Age**^#^ **(years) (median (range))**	55 (11–79)	62 (37–81)	68 (42–85)	61 (11–85)
**Sex^¶^ (female:male) (frequency)**	40:45	15:27	43:33	98:105
**VDP (%) (mean±sd)**	6.4±6.2	9.8±7.1	24.2±14.7	13.8±13.1
**VHI (mean±sd)**	11.9±2.9	13.2±3.4	16.5±3.7	13.9±3.9
**FEV_1_^+^ (% pred) (mean±sd)**	93.4±15.7	84.2±17.8	60.8±23.4	77.0±24.6
***T*_LCO_^§^ (% pred) (mean±sd)**	105±18.6	97.5±18.4	66.9±23.7	86.2±27.2
**CAT score***^ƒ^* **(mean±sd)**	9.2±7.4	12.3±8.3	19.0±9.2	14.4±9.5
**RV/TLC^##^ (%) (mean±sd)**	35.4±7.6	39.6±9.1	49.6±10.4	42.7±11.2

CAT: COPD assessment test; FEV_1_: forced expiratory volume in 1 s; RV/TLC: residual volume to total lung capacity; *T*_LCO_: transfer factor of the lung for carbon monoxide; VDP: ventilation defect percent; VHI: ventilation heterogeneity index. ^#^: Age missing for four patients. ^¶^: Sex missing for four patients. ^+^: FEV_1_ missing for 52 patients. ^§^: *T*_LCO_ missing for 53 patients. *^ƒ^*: CAT score missing for 48 patients. ^##^: RV/TLC missing for 53 patients.

These best performing models on the validation dataset were then evaluated on the testing dataset; the ViT model accurately classified 25/30 (83%) patients, achieving an F_1_ score of 0.82 (sensitivity 73%, specificity 93%, accuracy 83%), in comparison to the CNN model which achieved an F_1_ score of 0.62 (sensitivity 60%, specificity 67%, accuracy 63%). Testing set AUC values were computed, indicating that the ViT model produced a higher AUC than the CNN model (0.86 *versus* 0.71). Figure 1 depicts confusion matrices and corresponding accuracy metrics for the testing dataset for both the CNN and ViT models.

**FIGURE 1:**
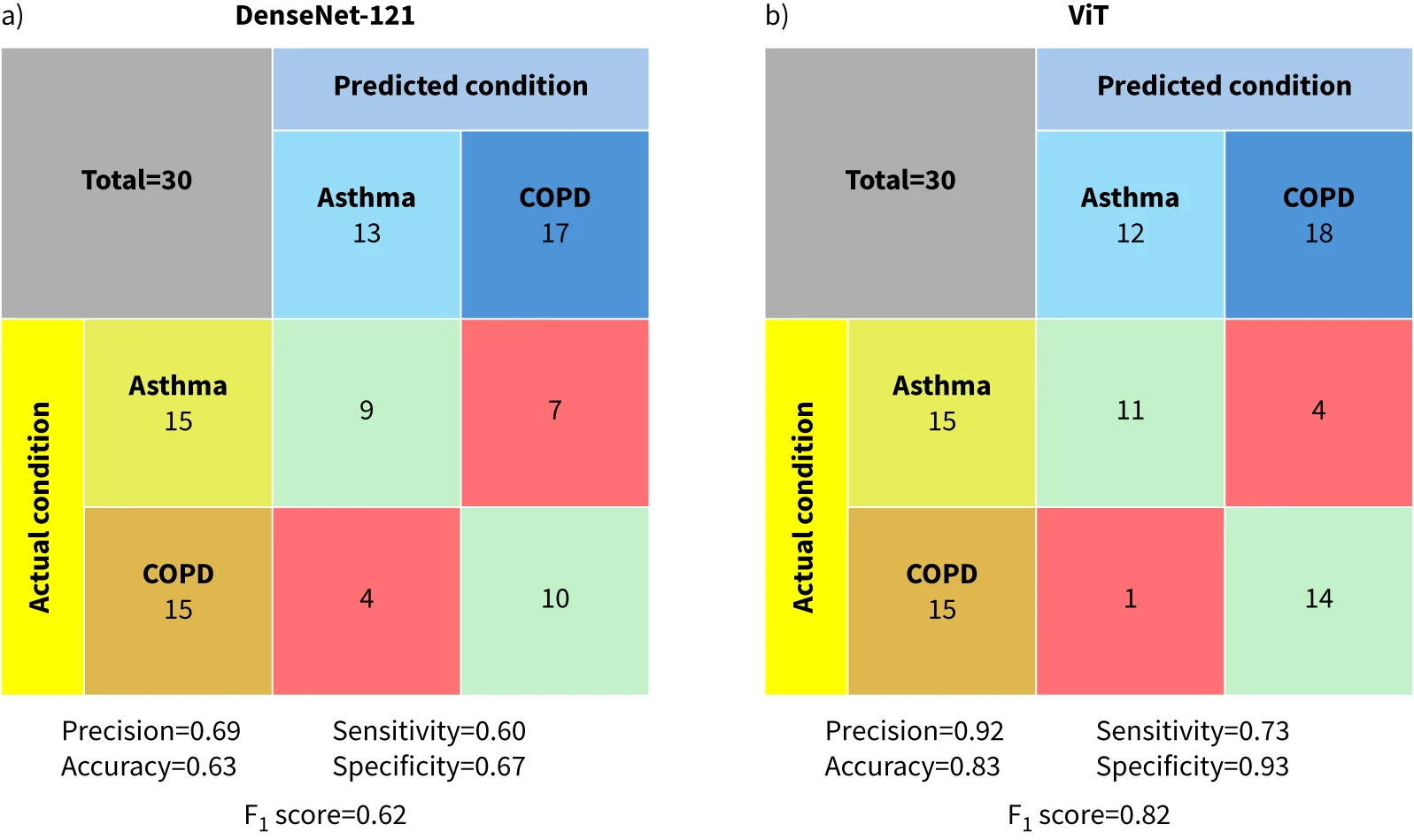
Confusion matrix and accuracy metrics for the testing set using a) DenseNet-121 and b) vision transformer (ViT) models.

### Occlusion sensitivity explainability

Alongside disease predictions, OccSens maps were generated, providing regional visualisations of areas of importance associated with the predicted classification. When classification was successful, regions of interest were usually within ventilated regions of the lung. Figure 2 displays example cases from the testing dataset with ground truth and predicted classifications, alongside prediction likelihoods and OccSens maps. In the five cases where ViT classification was unsuccessful, the asthma case misclassified as COPD had 29.1% VDP and the four COPD cases misclassified as asthma had relatively low VDP (5.3%, 5.4%, 6.4% and 8.6%).

**FIGURE 2:**
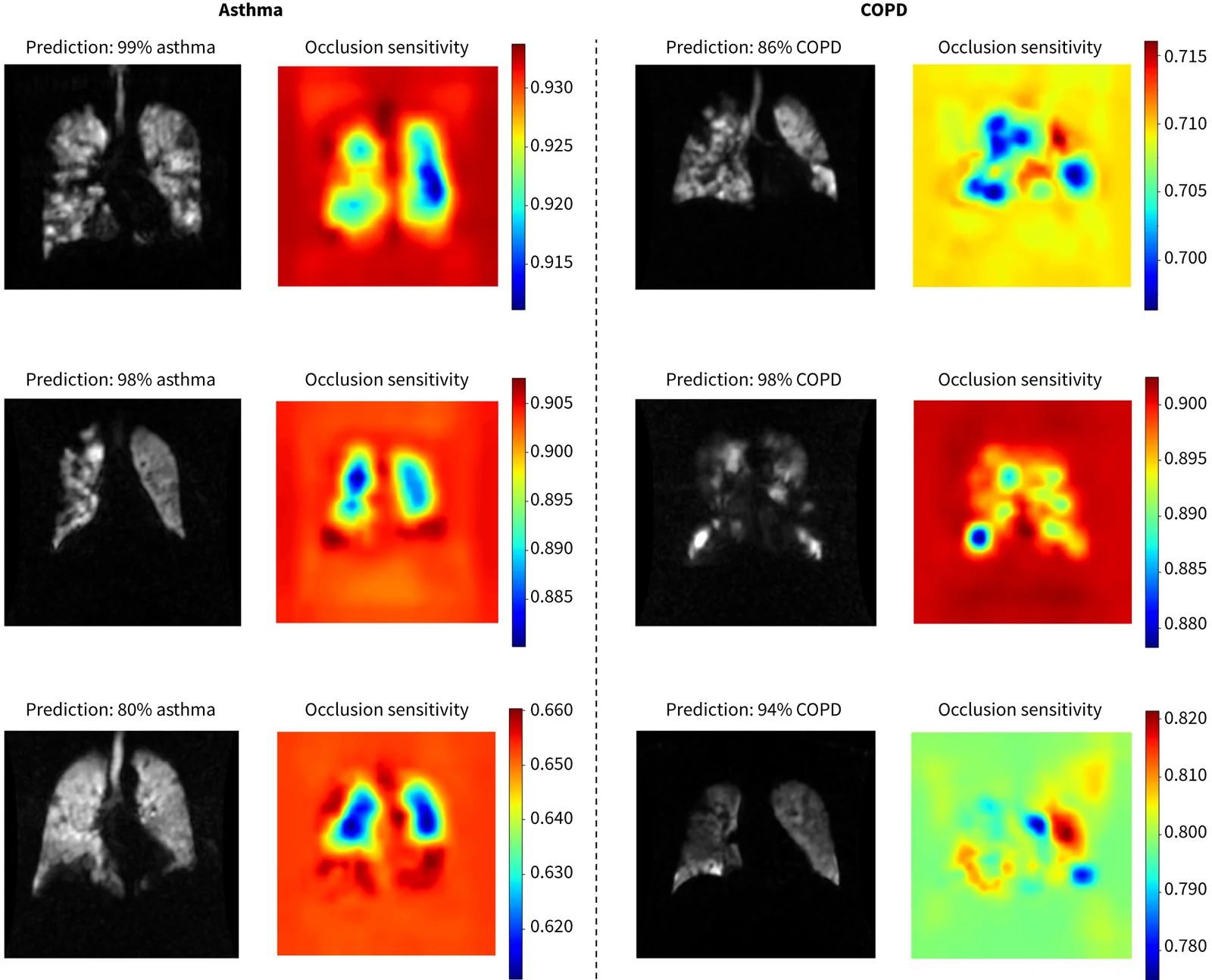
Six example cases from the validation dataset with ground truth and predicted classifications, alongside prediction confidence values. Xenon-129 ventilation (^129^Xe)–magnetic resonance imaging (^29^Xe-MRI) scans and occlusion sensitivity mapping (OccSens) maps are presented for a central slice. Blue areas indicate that the region is important in the predicted classification whereas red areas indicate regions with less importance to the network; this is measured by the colour bar which indicates the accuracy when this region was removed.

Based on a qualitative analysis of the resulting OccSens maps, we observed differences in the patterns of the AOIs for each diagnosis group; predominantly, asthma patients had more diffuse, elongated AOIs, whereas COPD patients had more focal, spherical AOIs. Due to these differences a secondary aim was constructed, namely to provide a single, normalised value for shape comparisons that effectively distinguishes between compact, spherical components and elongated or irregular structures. Figure 3 details a comparison of sphericity scores for all asthma and COPD cases within the dataset, demonstrating a significant difference in sphericity between disease groups (p=0.0003). Sphericity scores computed for the top-three connected components rather than the singular largest connected component demonstrated similar group-level difference (p=0.006). This highlights that group-level differences are not dependent on strict definitions of AOIs.

**FIGURE 3:**
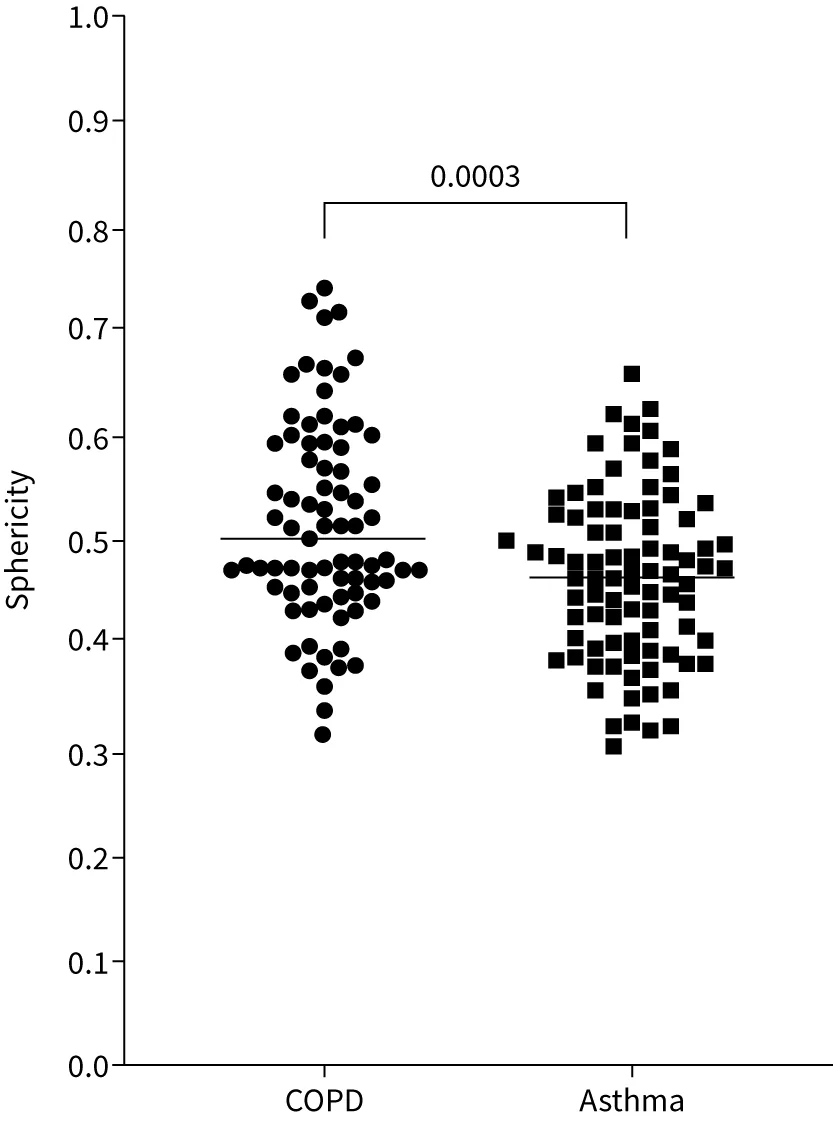
Comparison of sphericity scores for all asthma and COPD cases in the training, internal validation and testing sets.

The proposed sphericity score aims to provide a singular metric that is indicative of a continuum scale spanning from features of asthma to features of COPD. To this end, correlations between sphericity (ψ) and several lung function metrics were computed, as shown in figure 4.

**FIGURE 4:**
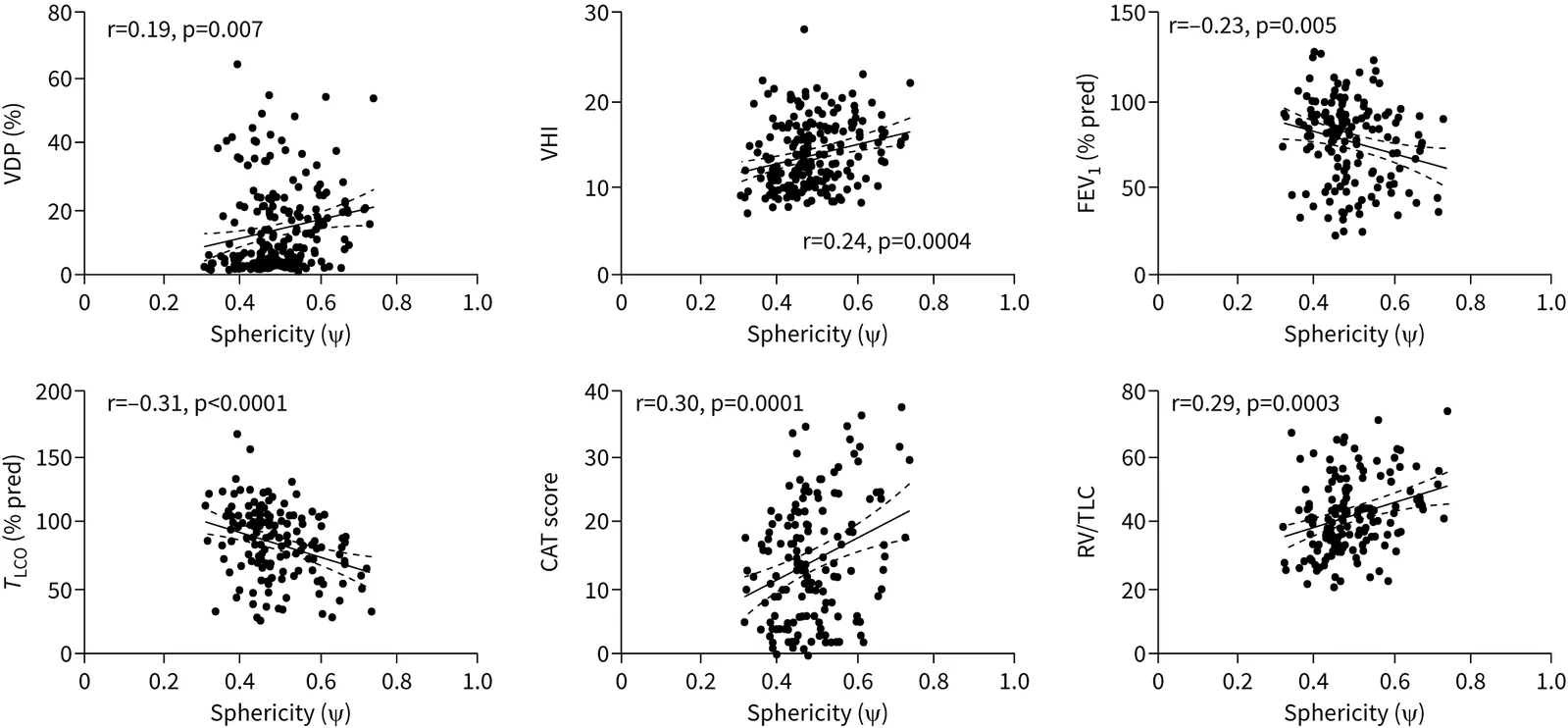
Pearson correlations between sphericity (ψ) and ventilation defect percent (VDP), ventilation heterogeneity index (VHI), forced expiratory volume in 1 s (FEV_1_), transfer factor of the lung for carbon monoxide (*T*_LCO_), COPD assessment test (CAT) score and residual volume to total lung capacity (RV/TLC).

### Overlapping features of asthma and COPD investigation

Asthma+COPD cases (investigation dataset) were classified using the ViT model. Most cases were classified as asthma patients (30/44) with a mean likelihood of 90.9%. In comparison, 14/44 asthma+COPD patients were classified as COPD with a mean likelihood of 86.3%. Nevertheless, in many instances the likelihood of one classification or the other was relatively equally split, representing cases with significant overlapping features. Figure 5 depicts asthma+COPD cases with a range of likelihood values. OccSens maps are displayed next to ^129^Xe-MRI scans, indicating areas of regional importance. Furthermore, the mean *T*_LCO_ z-scores were calculated for the likely-COPD and likely-asthma asthma+COPD cases. The likely-COPD group had a lower *T*_LCO_ z-score of −1.02±1.65 compared to −0.06±1.26 in the likely-asthma group, however this difference was not significant (p=0.09).

**FIGURE 5:**
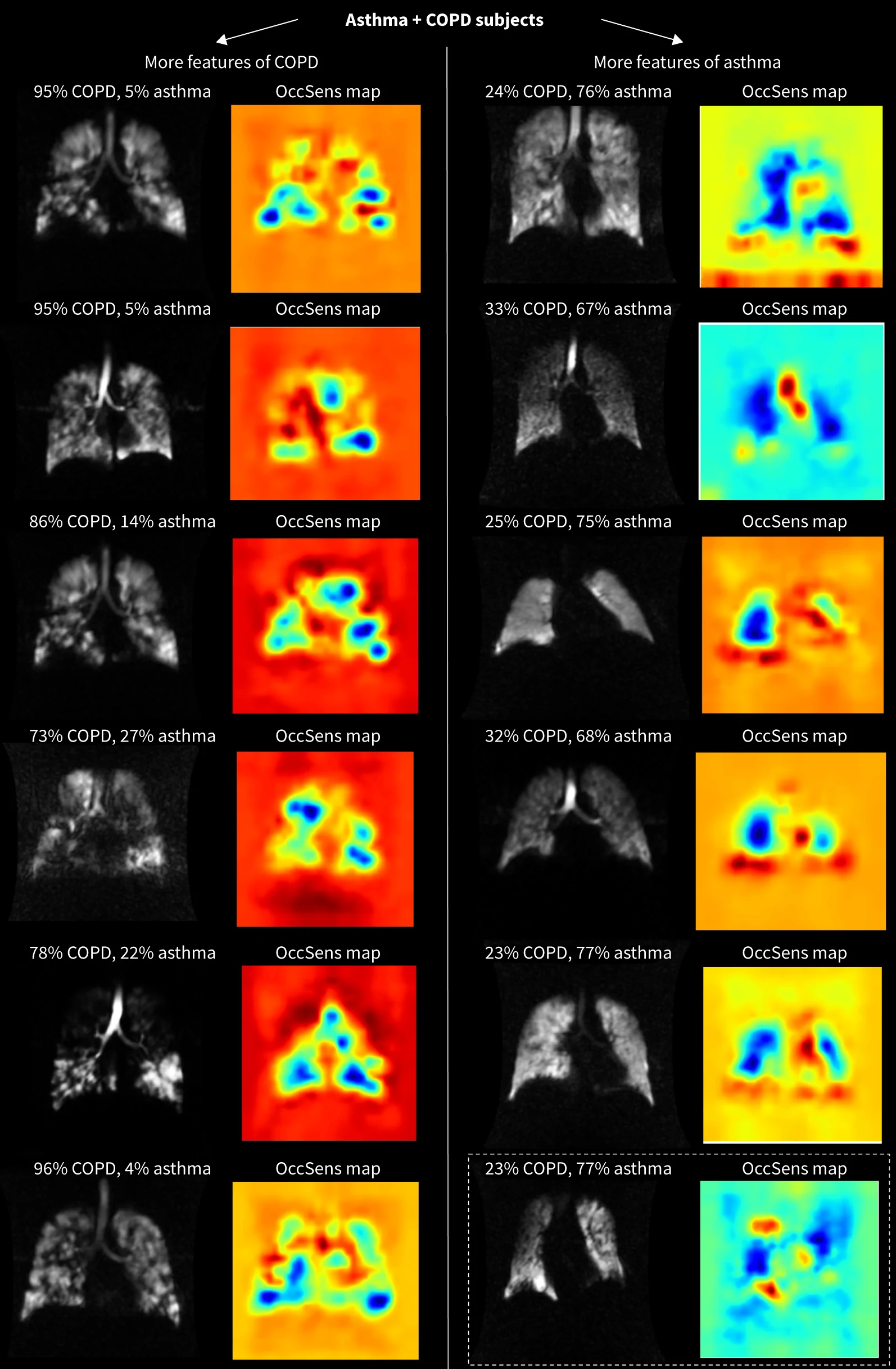
Example patients with asthma+COPD are shown. Xenon-129 ventilation (^129^Xe)–magnetic resonance imaging (^29^Xe-MRI) scans and occlusion sensitivity mapping (OccSens) maps are presented. Blue areas indicate that the region is important in the predicted classification whereas red areas indicate regions with less importance. Dotted line (bottom right) denotes a “failure” case with implausible areas of importance. Likelihood of asthma or COPD classification percentages are shown.

## Discussion

In this work, we utilise ^129^Xe-MRI scans from patients with asthma or COPD to build novel explainable deep learning-based classification networks. We demonstrate that a ViT outperformed a conventional CNN and is capable of accurately classifying asthma and COPD patients in 83% of cases, with matched mean disease severity in both disease groups. Furthermore, we employed an explainable AI technique known as OccSens to provide depictions of regional interpretability, facilitating insights into regional features associated with each classification group. OccSens maps indicate that background regions, including areas under the diaphragm, represent locations of low importance to the network. Conversely, some regions of ventilated lung parenchyma, including areas of high intensity, represent regions of high importance. The utility of explainability can be clearly visualised in figure 5, where a “failure” case shows areas of interest within the background, informing limited confidence in the prediction; this would be unknowable without explainable techniques.

There appeared to be differences in the OccSens patterns of interest for each diagnosis group; with larger, more diffuse AOIs in asthma and more focused AOIs in COPD. Consequently, we constructed a novel shape-based biomarker that aims to differentiate asthma-like and COPD-like features, derived from these OccSens maps. The calculated sphericity of important regions demonstrated significant differences between asthma and COPD patients. We observed weak but highly significant correlations between sphericity and reduced *T*_LCO_ (gas transfer), greater ventilation heterogeneity, more gas trapping and higher symptom severity scores, indicating that a higher sphericity score is consistent with features of COPD. However, it is also notable that the proposed sphericity metric is not strongly correlated with any specific pulmonary function test or ^129^Xe-MRI ventilation metric, indicating that the information provided by the sphericity of the OccSens maps is not strongly linked to or reliant upon conventional functional lung metrics. In future investigations, the proposed sphericity score could be correlated with longer-term outcomes as well as validated on a more extensive dataset to ensure the generalisability of the metric across diverse cohorts.

Matheson *et al*. [12] recently investigated the use of ^129^Xe-MRI to classify a range of different lung pathologies and healthy participants. Their study compares a range of two-dimensional CNN architectures, achieving an AUC of 0.78 when comparing asthma to all other classes present in the dataset; however, their dataset does not contain any COPD patients, instead including other pulmonary pathologies, such as cystic fibrosis. In this work, our primary aim was to tease apart closely related features in the heterogeneous and closely related conditions of asthma and/or COPD. To do this, we utilised a more complex 3D ViT model which generated improved performance, likely due to the inclusion of self-attention blocks and volumetric inputs which can better capture the complex ventilation heterogeneity of asthma and/or COPD conditions. Matheson *et al*. [12] employed an explainable AI technique known as Grad-CAM to explain their predictions [17]. Similarly, we propose an OccSens-based approach which, in our work, produced improved explanations compared to Grad-CAM++ [31] (see figure S1).

Defining both COPD and asthma has proven difficult. International guidelines produce lengthy definitions in order to do so and these have been subject to revision. In each case there has been increasing recognition of different endotypes, which seem pathophysiologically important in targeting therapy. This has led to some radical suggestions that airways disease be reclassified as eosinophilic and noneosinophilic instead of as asthma and COPD, pragmatically grouping diseases that share mechanisms that might be amenable to similar therapeutic manipulation [32]. Full characterisation of disease requires holistic assessment, but this study suggests that the division into COPD and asthma based on ventilation information from ^129^Xe-MRI is valid and may give important differences in symptoms and disease presentation, and evolution, which are likely to affect prognosis and may modulate therapeutic responses.

Quantitative computed tomography (CT) studies have found air-trapping and small-airway disease to be more severe in COPD than asthma [33, 34], and that emphysema is a feature of COPD only [34]. This is consistent with ^129^Xe-MRI and pulmonary functional test measurements, which additionally identified more heterogeneous ventilation and worse gas transfer in COPD than asthma [8]. Where available, these complementary measurements can help to distinguish asthma from COPD and therefore guide treatment. In the future, additional regional functional or structural information from ^129^Xe-MRI or CT, such as the emphysema distribution, could be included in deep learning models to classify asthma and COPD, and may improve outcomes further.

Our investigation had several limitations which may hinder clinical application. For instance, a relatively small dataset of 207 patients was used with validation and testing sets of only 20 and 30 patients, respectively. Moreover, the data was acquired at a single centre and therefore the generalisability of the proposed ViT may be limited. Furthermore, comparisons between CNNs and ViTs employed different parameters and implementations, such as input volume size, which may impact the validity of network comparisons. The comparison also indicates potential overfitting by the DenseNet-121 model which shows a significant reduction in AUC on the testing set compared to the ViT model which appears to generalise successfully (validation AUC 0.85 *versus* testing AUC 0.86). This reduction may demonstrate the improved robustness of the ViT architecture, despite the reduced input volume size compared to the CNN.

Additional limitations should also be considered. For a large number of patients (n=139), the diagnosis of asthma, COPD or asthma+COPD was assigned by a physician in primary care; this means that these diagnoses represent real-world treatment labels given to patients, but may also be inaccurate or revised based on more information. Moreover, the training dataset was not VDP-matched between asthma and COPD cases as this would have reduced the dataset to a size incompatible with deep learning approaches. The failure cases of the ViT approach being an asthma patient with relatively high VDP and COPD patients with relatively low VDP are likely a consequence of this. Also, whilst the testing set was VDP-matched between disease groups, the testing set was not matched for other functional imaging metrics, such as VHI. OccSens AOIs were defined based on an arbitrary 10% threshold with the largest connected component; whilst investigations were conducted by varying the number of connected components which demonstrated similar group-level differences, varying thresholds were not investigated and represent potential future robustness analyses. Further, the sphericity metric is theoretically volume–scale-invariant. However, AOI volumes were not formally controlled for as a factor in the group comparison, representing a potential limitation if the AOI has an abnormal volume.

### Conclusions

We demonstrate accurate classification of asthma and COPD patients, with matched mean disease severity in the testing cohort, from ^129^Xe-MRI ventilation scans using a ViT-based network. OccSens maps provide prediction explanations by showing regions associated with asthma and/or COPD, thereby increasing trust in the resulting classification. Quantitative evaluation of OccSens maps showed variations in shape-based regional patterns that may represent regional phenotypes of asthma and COPD. The proposed classification network and sphericity metric could potentially be used in the diagnosis of asthma and COPD, and to help determine if patients with asthma+COPD have more asthma-like or more COPD-like disease.

## Supplementary material

**Please note:** supplementary material is not edited by the Editorial Office, and is uploaded as it has been supplied by the author. Supplementary material 00234-2026.SUPPLEMENT
