# Supplementary material for: Explainable artificial intelligence-based classification of asthma and COPD from xenon-129 ventilation magnetic resonance imaging
Source: ERJ Open Res. 2026 Oct 19;12(5):00234-2026. doi: 10.1183/23120541.00234-2026 (PMC13718910; doi:10.1183/23120541.00234-2026)
Supplement: Supplementary file 1 [file 00234-2026.SUPPLEMENT.pdf]

Explainable AI-based classification of asthma and COPD from ^{129}Xe Ventilation MRI

Supplemental Material

Explainable AI frameworks

In the manuscript we utilise an explainable AI framework known as occlusion sensitivity mapping (OccSens) [1]. We further investigated another explainable AI technique known as GradCAM++ [2] which generates class activation maps of network layers within each deep learning model; GradCAM++ were produced using the open-source medical imaging framework monai 1.3.0 [3]. The specific details of GradCAM++ generation for the ViT and CNN networks are detailed in the following section.

GradCAM++

GradCAM++ builds upon a technique known as Grad-CAM [4] which produces coarse-grained localisations of regions of importance. The improved GradCAM++ produces more fine-grained maps by using the weighted combination of the positive partial derivatives of the activation function of the last convolutional layer [2]. Feature maps are produced with respect to a specific class score generating a weight map which corresponds to a visual explanation for the corresponding class label.

Comparison of GradCAM++ and OccSens visualisations

Figures S1 depicts comparisons of GradCAM++ and OccSens maps with corresponding ^{129}Xe-MRI scans, and prediction likelihoods for the same cases depicted in Figure 2; compared to OccSens maps, GradCAM++ provides a lower resolution depiction of regional areas of interest, limiting the potential regional insights that could be associated with each classification.

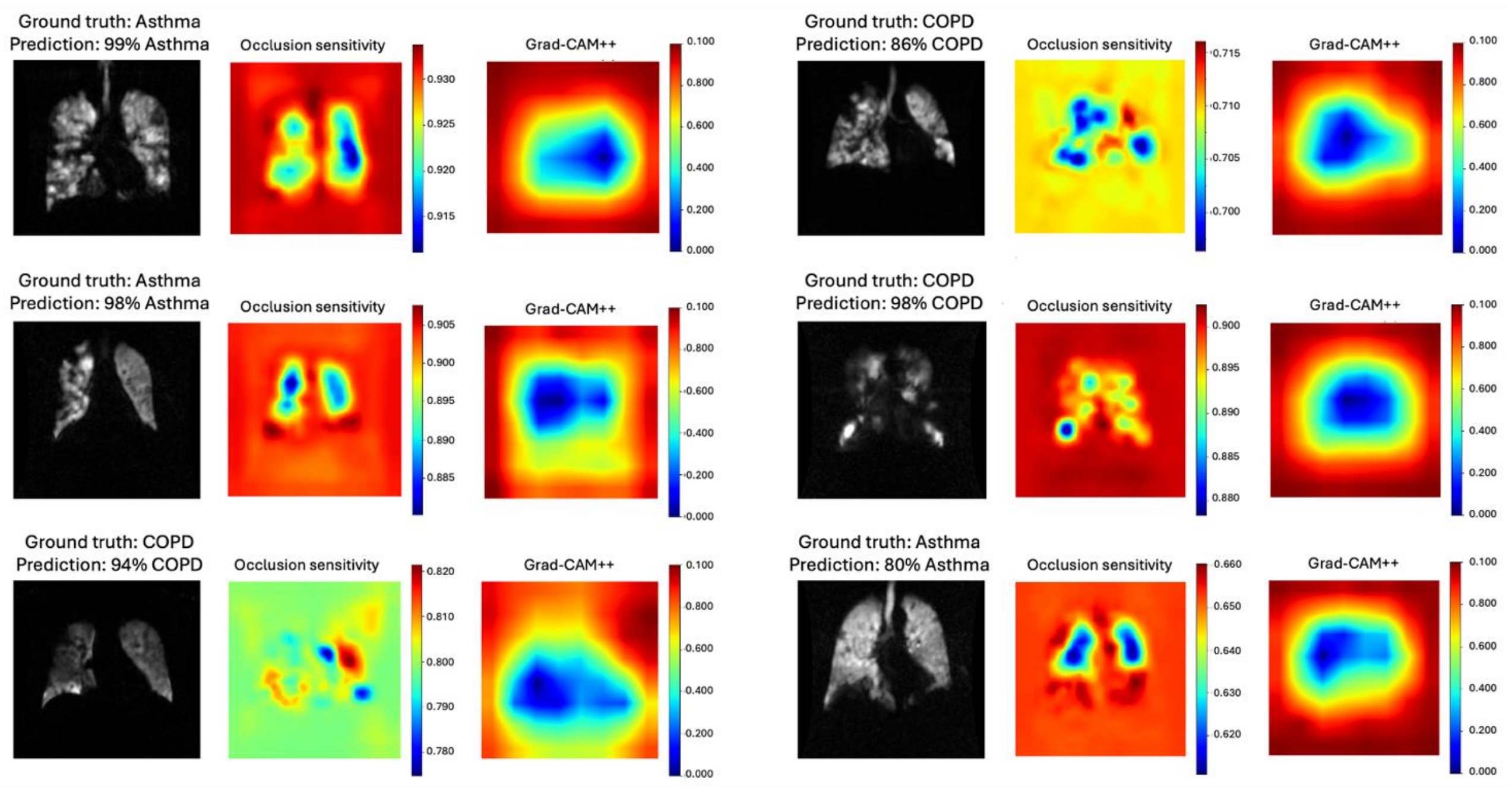

Figure S1. Six example cases from the validation dataset with ground truth and predicted classifications, alongside prediction confidence values. ^{129}Xe ventilation image, OccSens maps and GradCAM++ are presented for a central slice. Blue areas indicate that the region is important in the predicted classification whereas red areas indicate regions with less importance to the network.

References

1. Uchiyama T, Sogi N, Iizuka S, Niinuma K, Fukui K. Adaptive occlusion sensitivity analysis for visually explaining video recognition networks. *arXiv preprint arXiv:220712859* 2022.
2. Chattopadhay A, Sarkar A, Howlader P, Balasubramanian VN. Grad-cam++: Generalized gradient-based visual explanations for deep convolutional networks. In: 2018 IEEE winter conference on applications of computer vision (WACV); 2018: IEEE; 2018. p. 839-847.
3. Cardoso MJ, Li W, Brown R, Ma N, Kerfoot E, Wang Y, Murrey B, Myronenko A, Zhao C, Yang D. Monai: An open-source framework for deep learning in healthcare. *arXiv preprint arXiv:221102701* 2022.
4. Selvaraju RR, Cogswell M, Das A, Vedantam R, Parikh D, Batra D. Grad-CAM: Visual Explanations from Deep Networks via Gradient-Based Localization. In: 2017 IEEE International Conference on Computer Vision (ICCV); 2017 22-29 Oct. 2017; 2017. p. 618-626.
